# A methodological approach to intra-action reviews - application and adaptation of existing global guidance during the COVID-19 pandemic response in Ireland, 2021

**DOI:** 10.2807/1560-7917.ES.2023.28.13.2200475

**Published:** 2023-03-30

**Authors:** Eoghan O’Connor, Mary O’Riordan, Mary C. Morrissey, Niamh Dever, Cliodhna O’Mahony, Shem Romanowski, Máirín Boland

**Affiliations:** 1 Specialist Registrar Training Programme, Health Service Executive, Dublin, Ireland; 2 National Health Protection Service, Health Service Executive, Dublin, Ireland; 3 National Health Intelligence Unit, Research and Evidence, Health Service Executive, Dublin, Ireland; 4 Public Health and Primary Care Dept, School of Medicine, Trinity College Dublin, Ireland

**Keywords:** COVID-19, Intra-action review, pandemic response, public health, health protection, Ireland, recommendations, emergency preparedness

## Abstract

Many countries were under-prepared for the arrival of an emergency such as the COVID-19 pandemic. An intra-action review allows countries, systems and services to reflect on their preparedness and response to date, and revise their policies and approaches as needed. We describe the approach to undertaking an intra-action review of Ireland’s Health Protection COVID-19 response during 2021. A project team within National Health Protection developed a project plan, identified key stakeholders, trained facilitators and designed workshop programmes, employing integrated collaborative web tools. Multidisciplinary representatives participated in three half-day, independently facilitated workshops on challenges and solutions within specific response areas: communication, governance and cross-cutting themes such as staff well-being. An all-stakeholder survey sought further in-depth detail. Participants reviewed the ongoing pandemic response in terms of good practice and challenges and recommended implementable solutions. We customised our mixed-methods approach using existing ECDC/WHO guidance, producing consensus recommendations during Ireland’s fourth wave of COVID-19, with particular focus on pathways to implementation. Our adaptations may help others in formulating and customising methodological approaches. During an emergency, identifying and reflecting on good practices to retain, and areas for strengthening, with a clear action plan of implementing recommendations, will enhance preparedness now, and for future emergencies.

## Background

Despite public health pandemic preparedness efforts undertaken before the coronavirus disease (COVID-19) pandemic, many countries were under-prepared for a pandemic caused by a highly infectious respiratory pathogen such as COVID-19 [1]. An intra-action review (IAR) performed during a pandemic allows countries, systems and services to reflect on their preparedness and response to date and revise their policies and approaches as needed [1].

After-action reviews (AAR) are an established evaluation under the International Health Regulations (2005) framework following the official declaration of the end of a public health emergency event [2]. After-action reviews can identify areas for development; an AAR post West Nile virus transmission in Europe in 2018, for example, highlighted the ‘benefit of cross-sectoral and cross-disciplinary approaches’ to preparedness for such an outbreak in Europe [3]. When an incident is protracted, as in a pandemic, waiting until after the event precludes real-time feedback for immediate action. An IAR is carried out during the response, targets a defined period and has the additional aim of looking forward within the response to immediate possible solutions. An IAR is a key assessment of what has happened so far, to identify strategic priorities and can be used to exchange lessons learned between countries [4].

In Ireland, Health Service Executive (HSE) staff within Public Health, Health Protection division (hereafter referred to as Health Protection), continue to play a leading role in the pandemic response. In October 2021, the Health Threats Preparedness Programme, National Health Protection Service carried out an IAR of aspects of Ireland’s Health Protection COVID-19 response from January to October 2021 to (i) identify good practices and areas for strengthening, (ii) make recommendations to optimise the response to, and recovery from, the COVID-19 pandemic and (iii) strengthen the preparedness and response planning of the health system for the future. Of note, we addressed wider cross-cutting topics including communication, governance and staff well-being rather than traditional, strictly functional topics.

The IAR methodological process deserves focus, and this report describes the detailed application and adaptation of the European Centre for Disease Control (ECDC) and World Health Organization (WHO) guidance [4,5].

## Intra-action review process overview

**Figure 1:**
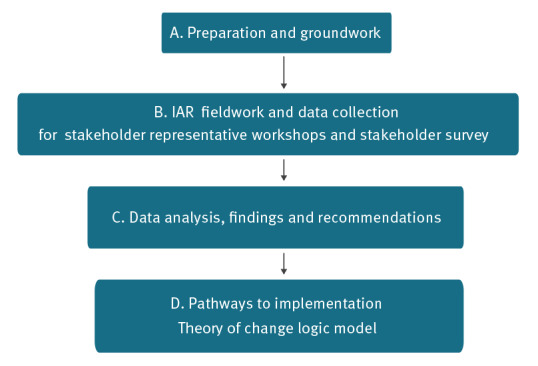
Overview of the main steps in the intra-action review process of the Health Protection COVID-19 response, Ireland, January–September 2021

We adopted a programmatic approach which is detailed below, comprising Part A: preparation and groundwork (five steps including planning meetings and an initial online questionnaire); Part B: the IAR fieldwork and data collection, including three stakeholder workshops (see Supplement S1, summary of IAR workshop: 10 component stages) with central project team meetings frequently held between workshops and a stakeholder survey; Part C: data analysis, findings and recommendation production; and Part D: pathways to implementation. An overview of the IAR process is shown in Figure 1.

IAR: intra-action review.

### Preparation and groundwork (Part A)

#### Defining the scope of the IAR

The IAR scope of focus was on Ireland’s Health Protection COVID-19 response between January and September 2021 during which the third and fourth waves of the pandemic occurred in Ireland. The review included the national and regional response, with a cross-discipline approach.

In June 2020, prior to the IAR, all national and regional Health Protection teams were invited via email to participate in an online questionnaire to identify the main issues in the public health response to the COVID-19 pandemic that required focus and improvement. The questionnaire design was based on key aspects of emergency response identified by WHO in their After-Action Review Toolkit [6] and adapted for the Irish context, with free text options. The top issues identified from the questionnaire were (i) communication within Health Protection, and with other stakeholders within and outside the HSE and with the public; and (ii) governance and decision-making processes. Other issues included surge planning, surveillance and staff well-being (through free text responses). Summarised findings for communication and governance are presented in Supplement S2.

The topics of communication and governance were chosen for the IAR pending consensus by voting at the first of the three stakeholder workshops (see detail below, Part B). These topics align to elements of the ECDC IAR COVID-19 response areas of risk and crisis communication, and emergency preparedness [7]. An emerging topic encompassing staff wellbeing and organisational culture arose repeatedly during the first IAR workshop and was included in the stakeholder survey, IAR scope and analysis.

#### Establishment of a central project team

A project team of six staff from Health Threats Preparedness, National Health Protection Service plus an independent facilitator identified through professional networks, was convened in August 2021 following planning meetings in July. The team had experience across health protection, public health emergency planning, project management, psychology and research methodology. From August to November 2021, the project team held regular meetings, often virtually, to collaborate and progress the work. Preparation intensified around the workshop periods, when the project team met up to three times in the week immediately prior to workshops to discuss, adapt and refine the workshop agenda and facilitation plan.

#### Experience-sharing meetings

Two meetings with the ECDC Emergency Preparedness and Response Unit in August and September 2021 were followed by a virtual learning exchange with Strengthened International Health Regulations and Preparedness in the EU (SHARP) [8] colleagues from the Robert Koch Institute, Berlin to discuss their IAR experience.

#### IAR approach

Based on the above experience-sharing, and ECDC/WHO guidance and templates, the project team developed a plan for the design and implementation of the IAR including issues such as methodology, scope and theme selection, facilitator recruitment and training, deliverables, design, timelines and resourcing requirements [4]. Roles and responsibilities for each member of the project team during the workshops were assigned and agreed on.

#### Stakeholder analysis

An analysis of key stakeholders was completed using a stakeholder engagement tool [9] and involved four key steps: (i) stakeholder identification: the central project team identified stakeholders based on their roles in the public health response and their ability to contribute to the purpose of the IAR; (ii) analysis: stakeholders’ interest in the IAR objectives, their input required, and their influence over the IAR objectives was recorded; (iii) mapping: the importance of the IAR objectives for each stakeholder was compared with their influence on the IAR objectives; (iv) planning: the level of engagement required from stakeholders in order to achieve the objectives of the IAR was discussed.

Forty participants from across each professional group within Health Protection at a national and regional level represented 318 stakeholders and took part in at least one of three workshops, with most attending sequentially (Table).

**Table:** Stakeholder groups represented at the three workshops during the intra-action review on COVID-19 response, Ireland, 2021 (n = 40)

Role of representative	Number of stakeholders participating in the workshops		
	Workshop 1 (September) n	Workshop 2 (October) n	Workshop 3 (October) n
Regional Directors of Public Health	2	2	1
Specialists in Public Health Medicine	7	8	8
Senior Medical Officers	3	2	3
Specialist Registrars in Public Health Medicine	1	1	2
Health Protection / Infection prevention and control nursing	2	3	3
Surveillance Scientists and Assistants	4	5	3
Administrators and managers including communications and IT within the Health Protection Surveillance Centre (HPSC)	4	4	5
National Public Health Leadership Team	6	4	5
**Total**	**29**	**29**	**30**

### Intra-action review fieldwork and data collection (Part B)

This IAR used a mixed-methods approach (qualitative and quantitative) consisting of three facilitated workshops with key stakeholder representatives, followed by a stakeholder survey.

#### IAR workshops

A series of three facilitated half-day virtual workshops were held during September and October 2021. We used the IT platforms Mentimeter (Mentimeter, Stockholm, Sweden) (https://mentimeter.com) interactive presentation software and Ideaboardz (Ideaboardz, Pune, India) (https://ideaboardz.com) collaborative whiteboard software to engage workshop participants (see Supplement S3-S6 for participants’ description of their experience of the pandemic (S3), participants’ expectations of the IAR process (S4), an example of potential solutions reached (S5) and a summary of IT platforms used (S6)).

The first workshop focused on an introduction to the IAR, stakeholder selection of the IAR topics, participant expectations of the process and challenges. The second and third workshops were dedicated to each of the two main IAR topics respectively (communication and governance). Workshops were hosted by the project lead, co-facilitated by the independent facilitator and project lead, and supported by the project team. Eight additional small-group facilitators were recruited through professional networks and trained for workshop 2 and 3. Each workshop followed 10 stages, shown in detail in Supplement S1: pre-workshop orientation; engagement; interaction; providing information; consultation and opportunity for deeper discussion; expression of participants’ opinions, experiences, perceptions; summarising and paraphrasing; partnership and managing expectations; evaluation and continuous quality improvement; reflection.

In keeping with the ethos of an IAR and with a continuous quality improvement approach, the project team incorporated their own reflections alongside feedback from the workshop participants and facilitators into the design and delivery of each workshop (see Supplement S7). Applying this iterative approach meant that facilitators had to adapt quickly to changing plans, sometimes in the middle of a workshop (for example where participants preferred to view raw breakout group quotes, rather than the presented summary/paraphrase, requiring agile IT use). Although challenging, this flexibility optimised the experience for workshop participants and also yielded richer contributions on the topics being discussed.

Participant feedback on aspects of workshop 1 was positive and is shown in Supplement S8.

#### Stakeholder survey, 2021

An online stakeholder survey was cascaded to all 318 colleagues working within national and regional HSE Health Protection in November 2021, seeking their views on the topics of communication and governance. This quantitative and qualitative survey was created using the online platform SmartSurvey (SmartSurvey, Tewkesbury, United Kingdom (UK)) (https://smartsurvey.co.uk) and contained 28 questions with multiple choice, free commenting and Likert scale answers. Information was also gathered on the respondents’ roles within Health Protection (regional/national), area of work (e.g. surveillance/nursing/medical/other) and duration of working within Health Protection.

The survey instrument was guided by outputs and discussions from the three IAR workshops, with some adaptation from the WHO Country COVID-19 IAR trigger question database [10]. The survey sought views on best practices and gaps in aspects of governance [11] and aspects of communication from two WHO pillar areas of risk communication: community engagement and infodemic management; and case management and knowledge sharing, in addition to staff well-being. Insights into ways to rapidly improve the response were sought.

The survey had a 38% (121/318) response rate, with responses received across all stakeholder groups. The resulting qualitative and quantitative outputs were collated and analysed by the project team using theoretical thematic analysis and descriptive statistical analysis (see Supplement S9, selected results of online survey).

### Data analysis, findings and recommendations (Part C)

#### Data analysis

The majority of data collected during this IAR was qualitative in nature, and analysed using topic-focused, theoretical thematic analysis [12]. This comprised seven steps including transcription, reading and familiarisation, coding, searching for themes, reviewing themes, defining and naming themes and finalising the analysis [13] (see Supplement S10). Software was relatively simple to use to collate and analyse themes (Dedoose version **9.0.17** **,** SocioCultural Research Consultants, Los Angeles, United States). Theme comparison between survey and workshop showed affinity/alignment between themes from both sources (Supplements S11-S12). Use of supporting quotes from participants and respondents contributed to a more powerful and evidence-based IAR.

#### IAR findings

A full report was compiled using bar charts, graphics and quotations to demonstrate the findings [14]. Selected findings are presented here for illustrative purposes.

**Figure 2:**
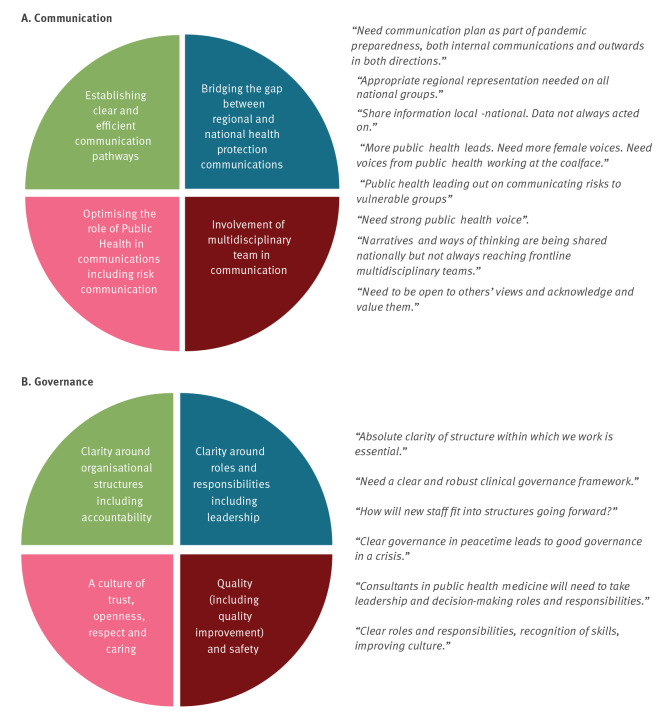
Subtopic areas for development within (A) communication and (B) governance, compiled from workshop 1 during the intra-action review on COVID-19 response, Ireland, January–September 2021

The priority topics of communication and governance were reaffirmed by stakeholder voting at the first workshop in September 2021 (29/29 agreed). For these topics, participants discussed challenges and areas for development using Ideazboard and in plenary, yielding four subtopics per topic. The subtopics for communication and governance (Figure 2) were used to direct discussion during each of the dedicated topic workshops.

The subtopics closely aligned with the stakeholder survey results (see Supplement S9). Furthermore, survey responses indicated the importance that respondents attached to communication, believing it to be an effective tool in health messaging and promoting behavioural change. Respondents highlighted inadequate opportunity to communicate directly with the public. Regarding governance and culture, they stressed the need for clear and documented decision-making. Two thirds (64/103, 62%) thought a culture of openness, trust and respect existed within their own unit.

Participants’ expectations of the IAR were gathered during the first workshop using Mentimeter (see Supplement S4). They hoped that the review process when implemented would lead to coordinated preparation, clear leadership and strategic planning, and strengthened communication within Health Protection.

Participants gave one word/phrase to describe their experiences to date of the COVID-19 pandemic using Mentimeter (see Supplement S3). Most frequent descriptors included: ‘exhausting’, ‘challenging’, ‘chaos’, ‘busy’, ‘overwhelming’, and ‘stressful’.

Progress achieved within Health Protection during the pandemic response was discussed in workshops 2 and 3 (what went well, enablers of high performance), and short and medium-term solutions were proposed. Participants also highlighted staff well-being and specific public health emergency planning as additional topics and urged early implementation of emerging recommendations from the IAR.

Three established key good practices in communication emerged during small group facilitated sessions, using Ideaboardz, followed by a plenary session: (i) having frequent, short, multi-disciplinary, departmental team meetings or huddles; (ii) establishing a dedicated communications officer within the Departments of Public Health; (iii) using stories to effectively communicate the public health message at a regional level, leading to behavioural change.

Three key good practices were identified in relation to governance during the health protection response to the COVID-19 pandemic: (i) the used of multi-disciplinary experience in the management of outbreaks; (ii) collaborative working between stakeholders; (iii) recognition of team member skills.

#### Recommendations

Six recommendations and 29 sub-recommendations were produced during three consensus meetings by the IAR project team using analysis of IAR consultation evidence, including themes and their frequency. The entire IAR report including recommendations was circulated in draft format to all workshop stakeholder representatives for consideration of accuracy and was accepted by all.

The six main recommendations to improve the Public Health Health Protection pandemic response currently and in the future include: (i) enhancing the Public Health communications function; (ii) optimising the use of information technology; (iii) capturing organisational knowledge; (iv) clarifying Public Health governance; (v) creating a culture that values staff and their contributions, with surge planning and staff wellbeing as key; and (vi) improvements in meeting processes (see Supplement S13).

Selected stakeholder survey results and full recommendations are presented in Supplementary materials (S9 and S13 respectively).

### Pathways to implementation (Part D)

Early consideration of implementation is vital, this being the ultimate aim of any IAR. In November 2021 we established an implementation group, including stakeholder representatives, with an independent senior management team chair, senior responsible owners for aspects of Public Health reform and those leading the development of Ireland’s first health protection strategy [15].

The implementation group reviewed and scored recommendations by implementation science methodology, using the Hexagon tool [16] to score each recommendation/need as low, medium, or high for its fit with: (i) current initiatives and policies; (ii) resource availability; (iii) evidence of effectiveness; (iv) intervention readiness; and (v) capacity to implement (see Supplement S14). A logic model was compiled to describe the theory of change, focusing on outcomes from the start of implementation [17]. The model covered (i) situation analysis (context); (ii) desired outcomes (changes to occur as a result of any interventions); (iii) key activities/tasks to achieve outcomes; (iv) resources needed; and (v) ongoing monitoring to assess progress. Recommendations were mapped and apportioned where relevant to the public health reform process, and the 2022–2027 Health Service Executive Health Protection Strategy [15]. The IAR and its recommendations were shared with the Public Health Reform Expert Advisory group which has a remit to ‘focus on identifying learnings from the public health components of the response to the COVID-19 pandemic in Ireland with a view towards strengthening health protection generally and future public health pandemic preparedness specifically’ [18].

## Conclusions

This intra-action review of the Public Health Protection response used established methodologies to assess good practice and challenges and to make recommendations to both improve the pandemic response and to improve preparedness for future such events. The Public Health response to the COVID-19 pandemic fits well with selection criteria for AAR/IAR. The aim of an IAR is organisational learning - ‘not to prove, but to improve’ [19]. While the topics of communication and governance were the chosen focus of this review, aspects of staff well-being, leadership and culture were consistently mentioned and thus incorporated. While it has been said that these types of reviews often reveal uncomfortable truths [19], what is important is the application of learning from this exercise.

Undertaken in Autumn 2021, during Ireland’s fourth COVID-19 wave, there was excellent engagement from stakeholders at workshops, with all representatives attending, and a 38% survey response rate from the 318 eligible staff. Thus, findings are a strong indication of Public Health staff’s views.

### Good practices

Good practices identified included regular multi-disciplinary team meetings (huddles) which are known to improve teamwork and process outcomes including clinical outcomes [20].

Regional departments lauded the establishment of dedicated communications officers, where present. Studies point out that dynamic infectious disease threats require specific approaches, strategically integrating social, online and traditional media for public health infectious disease communications [21] with leverage of strategies, including story-telling, to overcome any infodemic [22].

Collaborative, multidisciplinary working, with recognition of team member skills was a critical good practice identified during the COVID-19 response. The importance of the multidisciplinary approach has been noted internationally [23] with evidence of flexibility, interdisciplinary collaboration and rapid adaptation during the COVID-19 pandemic positively described elsewhere [24]. It is important to acknowledge the Public Health multi-disciplinary team input and adaptation as a key factor in enhancing the Public Health pandemic response.

### Areas for strengthening

Areas for strengthening included the establishment of clear and efficient communication pathways, externally and between regional and national functions. The role of Public Health in communications, including risk communications is vital, and stakeholders felt that the multidisciplinary team should be involved and represented. Expert opinion from an established, trusted source is a powerful tool [25]. The need to strengthen communication, including risk communication, is commonly found across after-action reviews [26].

Regarding governance, this IAR took place during a time of national Public Health reform with changing structures and functions. Stakeholders strongly identified the need for clear organisational structures, with clarity around roles and responsibilities sought. There was considerable positive commentary on leadership at regional and national level within Public Health.

Culture is a complex arena [27] and will require further elucidation. Public health is a female-dominated specialty in Ireland. The IAR participants sought gender balance in the media and at senior decision-making fora during the COVID-19 pandemic. Lack of gender balance is noted within the international COVID-19 response [28]. Insufficient attention to staff well-being and the impact of the pandemic on staff was described, in addition to inadequate staffing.

### Limitations

It could be commented that this review excludes other players involved in communicable disease control throughout the COVID-19 pandemic. However, WHO suggests a narrow IAR scope to assist focused learning [5]. Thus, this review confined the assessment to functions within the remit of Health Protection staff, which is appropriate.

The stakeholder survey response rate of 38% was considered acceptable given that staff were extremely busy as Ireland dealt with its fourth wave of the pandemic. When combined with the stakeholder workshops, findings were considered a robust indication of public health staff views.

Acting on lessons learned, at European level, ECDC promotes the use of after-action reviews as an evidence-based approach to assessing effective emergency response core capacities in real-life situations. Conducting intra or after-action reviews without meaningful learning from events can turn into a box-checking exercise. Learning from actual events requires overcoming a number of challenges [19], and how we apply findings is the crucial next stage. Implementation is the ultimate aim of any IAR. We presented recommendations with detailed sub-recommendations in clear action format. Following the establishment of a multidisciplinary implementation group, a number of the recommendations are already underway as part of the Public Health Reform Programme of Ireland and within the 2022-2027 Health Service Executive Health Protection Strategy. It has been commented that IARs/AARs are not widely shared with the people and divisions who could benefit from them [19]. It is vital that health systems use these findings to improve preparedness. Our insights may help other countries in formulating and customising methodological approaches. Identifying good practices to retain, as well as areas for strengthening and most importantly, a clear action plan of recommendations with paths to implementation, should enhance preparedness now and for future emergencies.

## Supplementary Data

741000 Supplementary Material
