# Supplementary material for: A methodological approach to intra-action reviews - application and adaptation of existing global guidance during the COVID-19 pandemic response in Ireland, 2021
Source: Euro Surveill. 2023 Mar 30;28(13):2200475. doi: 10.2807/1560-7917.ES.2023.28.13.2200475 (PMC10064645; doi:10.2807/1560-7917.ES.2023.28.13.2200475)
Supplement: Supplementary Material [file 22-00475_BOLAND_Supplementary_Material.pdf]

# Supplementary material for ‘A methodological approach to intra-action reviews - application and adaptation of existing global guidance during the COVID-19 pandemic response in Ireland, 2021’

This supplementary material is hosted by *Eurosurveillance* as supporting information alongside the article [A methodological approach to intra-action reviews - application and adaptation of existing global guidance during the COVID-19 pandemic response in Ireland, 2021], on behalf of the authors, who remain responsible for the accuracy and appropriateness of the content. The same standards for ethics, copyright, attributions and permissions as for the article apply. Supplements are not edited by *Eurosurveillance* and the journal is not responsible for the maintenance of any links or email addresses provided therein.

The supplementary material provides additional information on the following topics:


1. Summary of IAR workshop component stages: ten steps ........ 2
2. June 2020 survey findings ........ 6
3. Participants’ description of their experiences of the pandemic to-date (Workshop 1, Mentimeter) ........ 7
4. Workshop 1: Participants’ expectations of IAR (Selected direct quotes from Mentimeter) ........ 8
5. Workshop 3: Governance- potential solutions/ recommendations from plenary session ........ 8
6. IT platforms used to facilitate virtual workshops ........ 9
7. IAR Project Team de-brief post Workshop 1 (n=12) ........ 10
8. Participant feedback questionnaire post Workshop 1 (n=20) ........ 11
9. Excerpts from all-stakeholder online survey results ........ 12
10. Qualitative data analysis steps ........ 13
11. Word Cloud representing code application frequency from workshops (Dedoose) ........ 14
12. Word Cloud representing code application frequency from Stakeholders Survey (Dedoose) ........ 15
13. Six overarching IAR recommendations with additional sub-recommendations ........ 16
14. The Hexagon Tool ........ 18

## 1. Summary of IAR workshop component stages: ten steps

| Step | | Agenda Item | Description | Objective |
|---|---|---|---|---|
| I. | **Pre-workshop Orientation** | Pre-workshop background reading and instructions for participants | In advance of each workshop, participants were emailed information on background and scope of the IAR including a link to ECDC documents and WHO video explaining IARs. This email also included general 'housekeeping' information and advice re IT platforms to be used and internet connectivity. | • Participant familiarity and orientation with the IAR process<br>• Improved familiarity with IT platforms<br>• Reduced connection issues/ disruption during IAR workshops. |
| II. | **Engagement** | Welcome and Introductions | At each workshop, the project lead welcomed all and reminded participants of purpose of the workshop. 'Housekeeping' rules were outlined included the intention to run these workshops to the allotted time. Project lead also highlighted that the 'Chatham House Rule'[1] applied to all three workshops. Each participant was invited to speak and introduce themselves and the professional group that they were representing. At the first workshop, a short welcoming address from senior management was also scheduled. | • Orientation to purpose of workshop<br>• Participant awareness of others and groups represented<br>• Opportunity for all participants to speak to the full group for first time<br>• Building trust and reassuring in relation to confidentiality<br>• Respecting participants' time<br>• Visible buy-in from senior management |
| III. | **Interaction** | Icebreaker activity/ Opening Exercise | Workshop 1: Using Mentimeter, participants were asked for one word to describe their experiences of the COVID-19 pandemic to-date. They were also asked for their expectations of this IAR.<br>Workshop 2: Using Mentimeter, 'Describe in a word or phrase, what you would wish communication to be/ look like within Health Protection?'<br>Workshop 3: Using Mentimeter, 'Describe in a word or phrase what good governance looks like to you'. | • Provided early opportunity for interaction and participants' views to be shared<br>• Garnered expectations of participants at start of IAR<br>• Garnered participants' vision for what 'good' looks like in relation to IAR topics (communications and governance)<br>• Participants became familiar with Mentimeter |
| IV. | **Providing Information** | Didactic Presentation | Workshop 1: Short presentation on background and context to this IAR which included a video from 'Our World in Data' showing the epidemiology of COVID-19 in Ireland to-date and response milestones along the way. A short presentation on the results of the July 2020 survey was also included. | • Setting the scene for participants<br>• Providing background information and evidence base for IAR<br>• Feeding back and honouring the information previously garnered from participants in earlier |

[1] When a meeting, or part thereof, is held under the Chatham House Rule, participants are free to use the information received, but neither the identity nor the affiliation of the speaker(s), nor that of any other participant, may be revealed.

| | | | |
|---|---|---|---|
| | | Workshop 2: Short presentation capturing what we learned from the group in relation to Communications in workshop 1<br><br>Workshop 3: Short presentation capturing what was learned from the group in relation to Governance in workshop 1. | workshops and in July 2020 survey<br>• Re: presentation on July 2020 survey, this allowed project team to present the two main topics that arose from this survey (communication and governance) |
| **V. Consultation and opportunity for deeper discussion** | Voting on IAR topics and small group breakout sessions | Workshop 1: Using Mentimeter, participants voted on whether the topics of communication and governance (as highlighted in July 2020 survey) were still relevant. Participants were also asked to record on Mentimeter their thoughts now in relation to both topics.<br><br>Workshop 2 & 3: Following the short presentations capturing what was learned from the group in relation to Communications and Governance in workshop 1, participants were asked whether this was an accurate reflection and whether there were any gaps or omissions.<br>Participants were divided into pre-arranged smaller groups (5-7 participants) on two occasions during each workshop. The small groups comprised of a mixture of representatives. Each small group was allocated one sub-topic within Communications/ Governance. They were asked to discuss and record 'what went well, and why' and 'what needs to be improved, and how can we do that' (taking into consideration any emerging issues on the horizon) in relation to each of the sub-topics. The latter question garnered a list of potential solutions that could be implemented to improve performance. These breakout sessions were facilitated and 'Ideaboardz' whiteboard IT platform was used by participants to capture their views. | • Sense-checking with participants<br>• Participants validated that topics of communication and governance were still relevant by consensus<br>• Qualitative analysis of participants' views from Ideaboardz in relation to communications and governance yielded sub-topics under each topic for further exploration<br>• Breakout sessions provided opportunity for participants to share more detailed views on focused areas; to acknowledge and harness the good practices that had been used; and to prioritise the solutions.<br>• Opportunity for small group participants to assemble their collective views in real-time using collaborative white board software.<br>• Amalgamation and display of all small group views<br>• All voices/comments captured |
| **VI. Expression of participants' opinions, experiences, perceptions** | Feedback from small group breakout sessions and facilitated group discussion | Workshop 1: Following consultation via Mentimeter, a plenary group discussion was facilitated where participants were encouraged to share their views (in general) on the topics of communications and governance within Health Protection.<br><br>Workshop 2 & 3: One rapporteur was nominated by each small group to feed | • Building trust<br>• Feeding back, sharing and honouring the information provided during the breakout sessions<br>• Allowing all voices and stakeholder groups to be heard |

| | | | |
|---|---|---|---|
| | | | back the main points and prioritised solutions from the breakout sessions. Participants were reassured that all the information captured on Ideaboardz would be included in the data analysis. This was followed by a facilitated plenary group discussion. The lead facilitator paid particular attention to ensure that all stakeholder groups were represented and heard during these plenary discussions, calling on representatives to speak as necessary. | • Opportunity to expand on and share salient points from small group breakout sessions<br>• Opportunity to raise any new/ outlier sub-themes |
| **VII.** | **Summarising and Paraphrasing** | Closing the facilitated group discussion | Lead facilitator and project lead summarised and reflected back the conversations during the plenary group discussion. | • Sense-checking with participants<br>• Demonstrating accurate understanding and interpretation of views of the group<br>• Building trust |
| **VIII.** | **Partnership and managing expectations** | Next Steps | Short presentation at the end of each workshop outlining<br>• Next Steps<br>• What will be covered in the next workshop<br>• What participants can expect from the project team before the next workshop e.g. additional information via email<br>• Expectations of participants before the next workshop e.g. engage with their stakeholder groups to garner views | • Clarity on roles and responsibilities of both the project team and participants<br>• Building sense of partnership in this IAR process<br>• Managing expectations of participants |
| **IX.** | **Evaluation and continuous quality improvement** | Feedback from participants and Close of workshops | At the end of each workshop participants were asked for their immediate reactions (using Zoom emojis) to the workshop, and given a link to a post-workshop online survey to provide early feedback to the project team on that workshop (link sent out in email also after event). This feedback was analysed and used to inform the design of the subsequent workshop.<br><br>In some cases, participants gave feedback throughout the workshops, and where possible the project team aimed to implement this feedback immediately e.g. on one occasion the project team changed the method of voting and prioritising from Mentimeter to Ideaboardz based on participants' stated preferences, as it was more conducive to capturing accurate representation of their views. At the close of each | • Opportunity for participants to give feedback on the IAR process and running of workshops<br>• IAR team listening to and honouring that feedback<br>• Opportunity for project team to continuously adapt and improve methodology employed<br>• Building sense of partnership in this IAR process |

| | | | |
|---|---|---|---|
| | | workshop the project lead thanked participants for their time and contributions. | |
| **X. Reflection** | Debrief | Following each workshop the project team and any additional facilitators held a de-brief session on the workshop exploring:<br><br>• What went well<br>• Challenges encountered<br>• What needs to be changed for the next workshop<br><br>In some cases, this reflection also took place during the workshops. For example, during workshop 1 the schedule was altered to include a longer facilitated group discussion to give participants an opportunity to engage and voice their opinions. This was then built into the schedule for workshop 2 and 3. | • Opportunity for project team to acknowledge and harness the good practices<br>• Opportunity for project team to continuously adapt and improve methodology employed |

## 2. June 2020 survey findings

**Purpose**

The purpose of the 2020 online survey by the National Office of Health Protection was to highlight key issues in the public health response to the COVID-19 pandemic that required further analysis and discussion.

**Method**

The survey design was based on the key aspects of an emergency response identified by WHO in their After-Action Review Toolkit and adapted for the Irish context. Free text boxes were incorporated into the survey to allow participants to provide reasons for their chosen responses.

**Aspects of the pandemic response to review:**

- Communication: within Health Protection, with other stakeholders both within and outside the HSE, and with the public
- Governance and decision-making processes
- Surge capacity
- Surveillance Systems

**Communication key areas to review:**

- Development of cohesive relationships between national and regional decision-making forums.
- Development of formalised and streamline Information flow between national and regional functions.
- Development of priority setting with clarifications on roles and responsibilities.

**Governance key areas to review:**

- Clarity of roles, responsibilities and accountability in relation to national and regional forums and national-regional interface.
- Clarity of the decision-making processes, the reporting structures and the national-regional interface.
- Clinical and data governance clarity particularly around testing, interpretation, results, contacts and data provision to various stakeholders.

3. Participants' description of their experiences of the pandemic to-date (Workshop 1, Mentimeter)

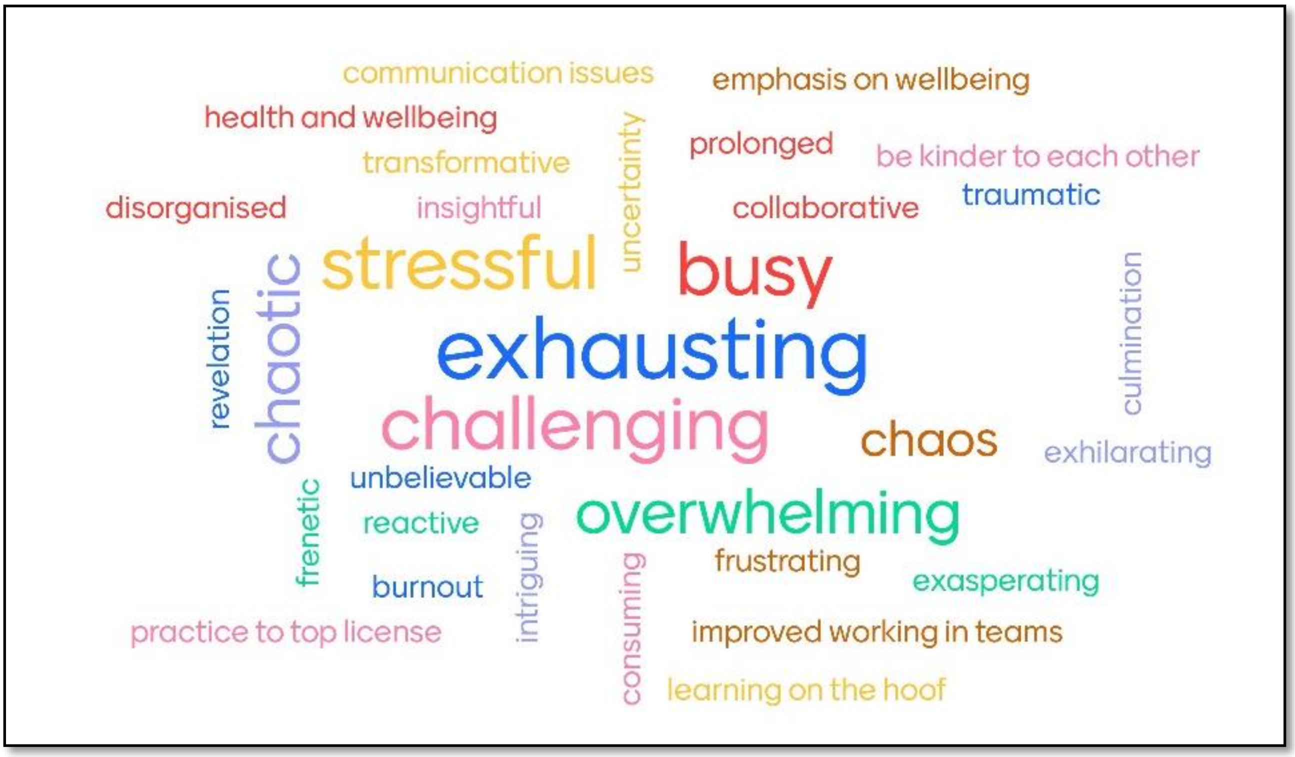

## 4. Workshop 1: Participants' expectations of IAR (Selected direct quotes from Mentimeter)

| **Participants were asked: What would you like to get from this intra-action review? What are your expectations?** | |
|---|---|
| **Implementation**<br>- The future response outlined and resourced<br>- Implement solutions and recommendations<br>- Cut through red tape<br>- Commitment to early implementation<br>- Follow through on any recommendations | **Communication**<br>- Clear communication and understanding of Public Health role<br>- Develop guidance and awareness on roles of Public Health<br>- Improved communication |
| **Leadership and co-ordination**<br>- Certainty and direction going forward<br>- Clear direction for future pandemic planning<br>- Coordinated pandemic response nationally<br>- Clear plan for comprehensive Public Health response to next phase of pandemic<br>- A series of actions for the future plan<br>- Structured way of working going forward<br>- Improvements to work better together | **Improvement**<br>- Learning and focus<br>- Ensure we learn from this response, valuing Public Health<br>- Flexible digital tools and improved data systems<br>- Realistic, well supported actions for preparation for future pandemic response<br>- Integrated information systems that address all needs for information exchange, ad-hoc and routine scheduled data flows.<br>- Building on what we have learnt about the important of multidisciplinary team, recognition of specific skill-sets |

## 5. Workshop 3: Governance- potential solutions/ recommendations from plenary session

Six potential solutions related to Governance were identified by participants during small group facilitated sessions followed by a plenary. These are listed below in no particular order of priority.

I. Ensure clearly defined roles and responsibilities

II. Develop standard operating procedures

III. Use evidence-based guidance developed by the appropriate body

IV. Contact tracers should be working on site/within departments

V. Introduce consistent administrative support for national meetings

VI. Clarify the role of Health Protection Nursing and improve induction of new staff

## 6. IT platforms used to facilitate virtual workshops

| Requirement | IT Platform/ Software used |
|---|---|
| Virtual meeting software with capability for breakout rooms and chat function | Zoom (Zoom, 2022) |
| Virtual voting and interactive sharing of views | Mentimeter (Mentimeter, 2022) |
| Virtual collaborative whiteboard | Ideaboardz (Ideaboardz, 2022) |
| Online Survey | Smart Survey (Smart Survey, 2022) |
| Quantitative analysis of data | Smart Survey and Microsoft Excel (Microsoft Corporation, 2018) |
| Qualitative analysis of data | Dedoose[2] (Socio Cultural Research Consultants, 2021) |

[2] Dedoose Version ***9.0.17***, web application for managing, analyzing, and presenting qualitative and mixed method research data ***(2021)***. Los Angeles, CA: SocioCultural Research Consultants, LLC www.dedoose.com.

## 7. IAR Project Team de-brief post Workshop 1 (n=12)

| What went well? | What needs to be improved? | Actions to follow |
|---|---|---|
| • Flexibility<br>• Adaptability in a short space of time.<br>• Mentimeter worked well<br>• Smooth, preparation went well<br>• Pace was good<br>• Independent facilitation worked well<br>• Multidisciplinary collaboration worked well the way we captured comments was exactly how they said things- I think (participants) felt safe<br>• Remodelled the process for our needs<br>• Core team's commitment to the outcome came across very well<br>• People had a chance to talk about their experience and they have a chance now to go over the whole year, discuss it, put it on paper and implement<br>• It went well from the introduction, good timing and pacing, voices were clear<br>• Input from leadership was crucial<br>• Sound and room set-up were good<br>• Chat – engagement was good<br>• The plenary went well<br>• Slides – colours and design were appropriate, superb<br>• Flexibility was accommodated | • Full presentation on one platform is better<br>• Comfort – safety to speak and express views<br>• Preparation was short and there should be more dry runs<br>• Practising should be done more often<br>• Slide changes on Mentimeter are difficult – final slide set draft should be submitted earlier than a day before<br>• Waiting room function in Zoom should be double checked<br>• A plenary should include someone from every professional group who should be invited to speak. | • Planning next event<br>• Keep the momentum going<br>• Scheduling of meetings to prepare<br>• Mapping out<br>• Well-being seems more important to some than communication<br>• Team to draft a schedule and work with WHO materials<br>• Add in colleagues who agreed to facilitate<br>• Stakeholders should not be facilitators<br>• Thank you letter to participants, slides, participants list, post-event survey<br>• 1 hr session to review Stakeholder survey and use workshop data to inform it.<br>• Collate all the feedback into one document for team's review |

## 8. Participant feedback questionnaire post Workshop 1 (n=20)[3]

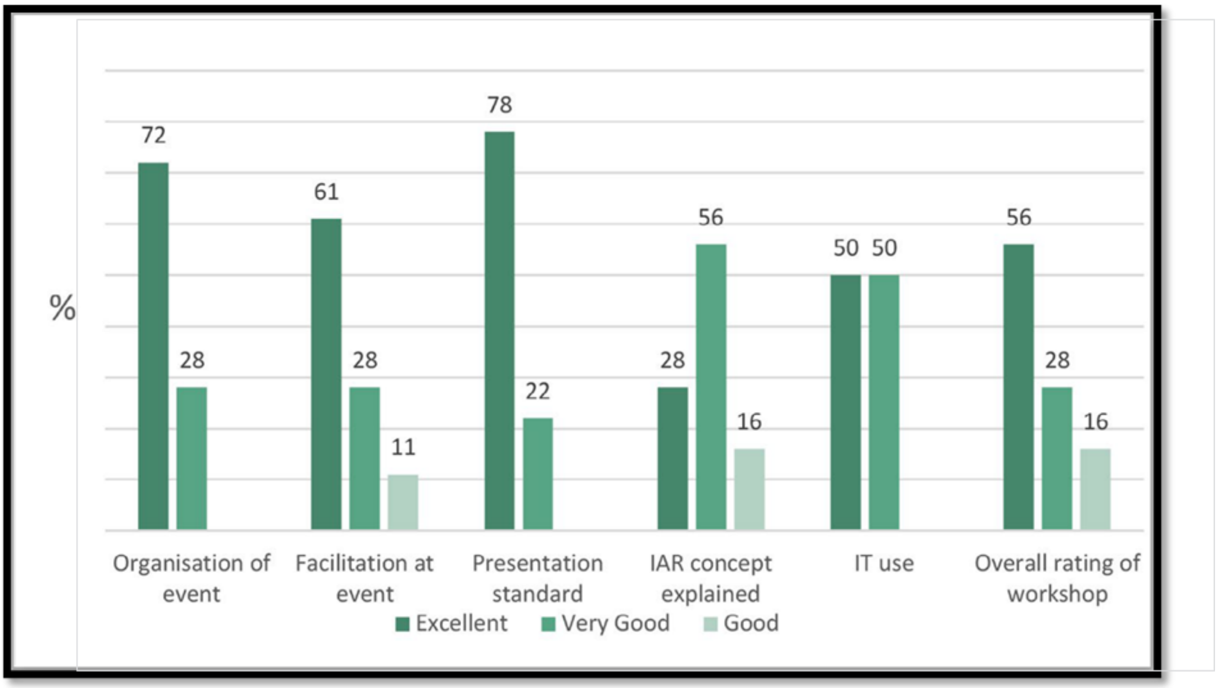


| **Do you have any further comments in relation to the event itself? (n=20; 7 comments)** |
|---|
| Very interesting methodology |
| Well done to all of the organisers. It was evident that a lot of work had been put into organising this IAR. |
| Very well organised and thought provoking. Very pleased to have the opportunity to attend and learn from experience and hear the input from a wide range of stakeholders within PH. |
| Good use of Menti, makes the engagement process much easier. Possibly, having access to questions prior to IAR would allow a more considered response which might help improve the outputs from the IAR |
| Over-represented medical staff -other disciplines are less well represented. The Mentimeter tool is useful as it allows other disciplines to contribute which is not that easy in a group dominated by medical staff. |
| Leaders and those working nationally, or on national groups worked really hard at a very high level….in this forum, I think it was important for them to listen… |
| Honestly, I came away from it feeling invigorated.<br>I've already learned things about excellence in preparation and organisation of such an event.<br>I look forward to contributing further.<br>I predict that output will be clear, strong and realistic, and will reflect the urgency that's needed for implementation. |

[3] 'Fair' and 'Poor' were options for participants but were not ticked

## 9. Excerpts from all-stakeholder online survey results

**Summary of survey results on communication**

- Most respondents indicated that internal communication messages are well developed and clear, and that relevant information is received. Channels for internal communication need strengthening.
- Most respondents indicated that messages sent externally are generally felt to be clear and accurate.
- External communication channels need development in the areas of receipt and cascade of external information to those within health protection.
- Survey responses indicate the importance that respondents attached to communication, believing it to be an effective tool in health messaging and promoting behavioural change.
- Respondents highlight inadequate opportunity to communicate directly with the public.
- Overall, feedback did not seem to be sought or acted upon to improve messaging/communication during the period.

## 10. Qualitative data analysis steps

| | |
|---|---|
| **Transcription** | All workshop discussions were transcribed into text and anonymised. The qualitative data from the stakeholder survey, which was partially anonymised but did include descriptors, was collated. All files were uploaded to Dedoose[4]. |
| **Reading and Familiarisation** | Each file was read/analysed once without applying codes to gain an understanding of the general discussion. |
| **Coding** | The lead data analyst (IAR team member) coded each file using Dedoose[5] web application by searching for quotes or phrases pertaining to the over-arching topics. Open codes were generated and modified during the data analysis, and double checked. |
| **Searching for themes** | Codes were analysed and grouped together where appropriate to seek emerging overarching themes. There were also additional themes that arose solely from the stakeholder survey (eg leadership, culture). |
| **Reviewing themes** | The data analyst critically analysed the quotes pertaining to each theme to decide if the theme accurately reflected the coded quotes i.e. did the data support the theme. |
| **Defining and naming themes** | Themes were named as a descriptive phrase/sentence. Quotes pertaining to each individual theme were downloaded together and these quotes were grouped together in the final report to document the evidence supporting the thematic analysis. Each theme was discussed with direct reference to participant quotes. The prevalence of themes was considered important, with themes recurring more frequently and by various participants given more weight in the context of the analysis. |
| **Finalising the analysis** | Critical appraisal of all coded quotes identified the key messages being conveyed by participants of the IAR. These key messages either related to what went well during the Public Health response to the pandemic or to areas that could be improved including suggested solutions. Additional themes were included in the thematic analysis, with reference to supporting quotes. |

[4] Dedoose Version ***9.0.17***, web application for managing, analyzing, and presenting qualitative and mixed method research data ***(2021)***. Los Angeles, CA: SocioCultural Research Consultants, LLC www.dedoose.com.

## 11. Word Cloud representing code application frequency from workshops (Dedoose)

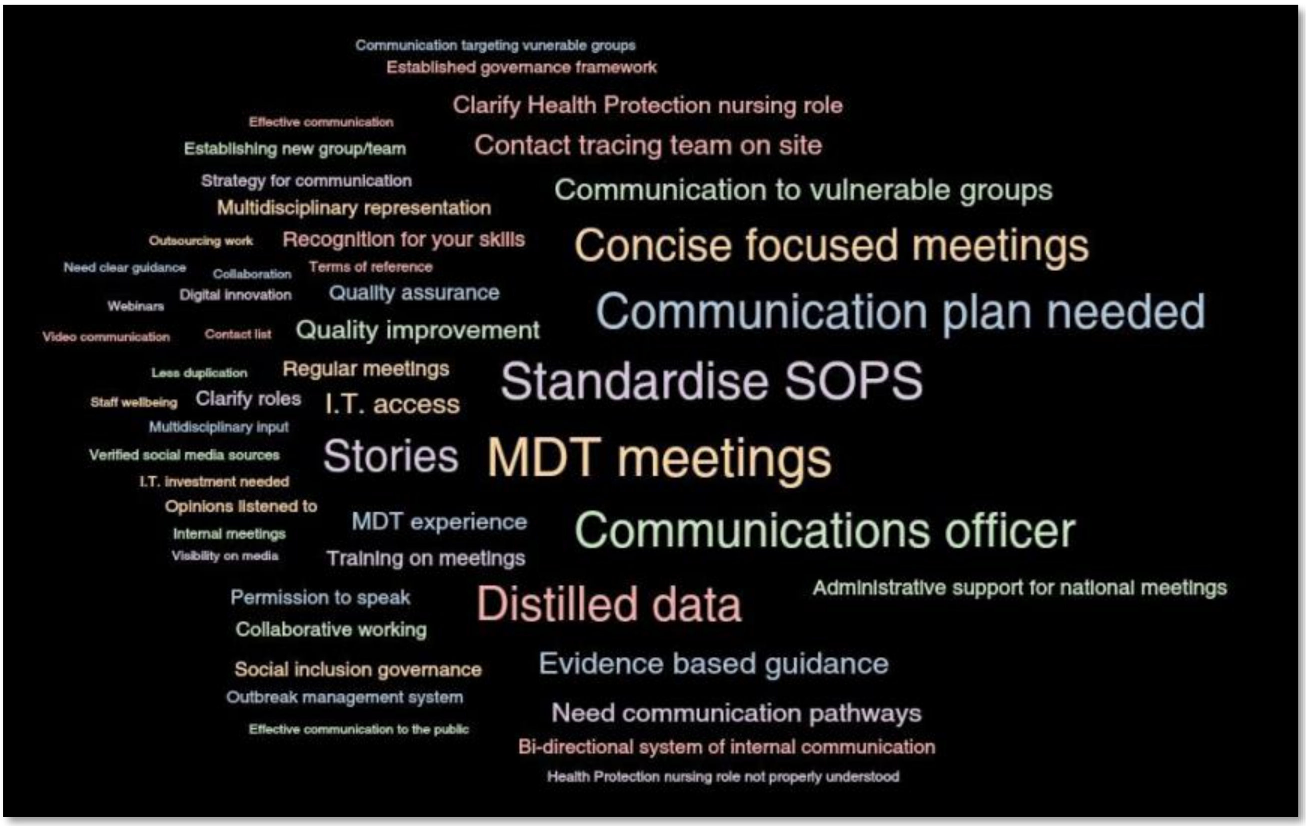

## 12. Word Cloud representing code application frequency from Stakeholders Survey (Dedoose)

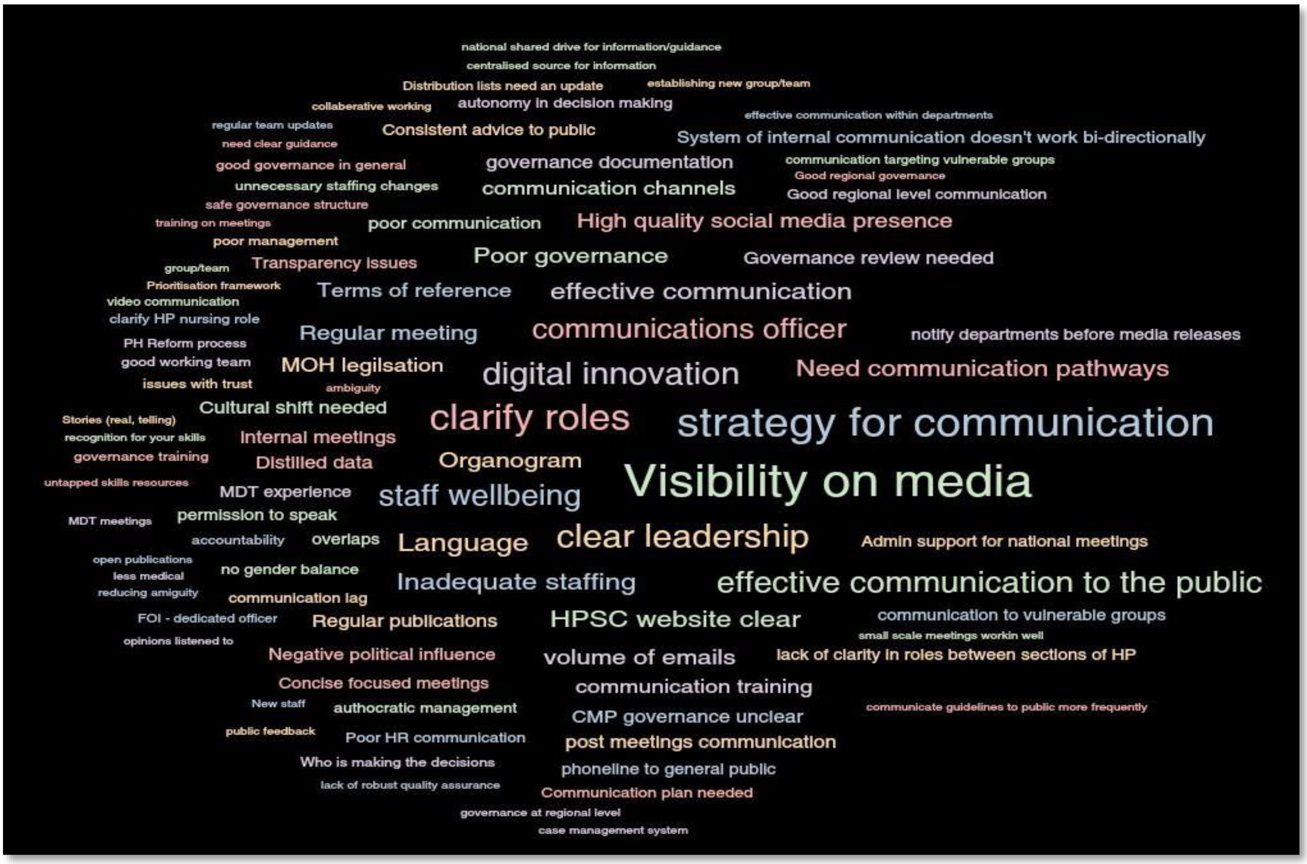

## 13. Six overarching IAR recommendations with additional sub-recommendations

Potential solutions were gathered from the IAR workshops and the stakeholder online survey, categorised by the IAR project team and catalogued into six overarching recommendations with additional sub-recommendations which are presented below. IAR recommendations are intended to be readily implementable in the short and medium term; the analysis also yielded some longer-term recommendations which are included below.

1. **Enhance the Public Health communications function to enable, support and promote the use of Public Health expertise.**
    a. Develop a Public Health communications operating model.
    b. Establish a network of communications officers for Health Protection working across regional and national levels.
    c. Promote and enable increased visibility of Public Health subject matter experts across various platforms including media.
    d. Strengthen risk communication capabilities including training, education and support for staff.
    e. Develop a central resource of standardised communication resources tailored to specific vulnerable groups.

2. **Use enhanced and up-to-date information technologies to optimise Health Protection communications.**
    a. Identify opportunities to integrate data systems and streamline information flow between departments (regionally and nationally) so the systems are capable of fully supporting staff to manage routine and ad hoc events.
    b. Provide a dedicated case and incident management system.
    c. Strengthen general IT support and resources for staff.
    d. Harness up-to-date IT capabilities to enable timely and appropriate dissemination of information.

3. **Capture organisational knowledge**
    a. Develop, review and maintain an inventory of committees; terms of reference, governance and reporting structures including for emergency events.
    b. Develop and maintain an accessible overview of organisational details; organogram(s), staff roles, responsibilities, reporting relationships, contact details and communication pathways including for emergency events.
    c. Strengthen the production and structured dissemination of standardised operating procedures and evidence based clinical guidance.
    d. Use organisational knowledge to inform preparedness and planning strategy
    e. Ensure that appropriate Health Protection technical expertise is included at senior level decision-making fora.

4. **Clarify Public Health Protection governance in the context of current Public Health reform programme.**
    a. Review and clarify leadership, preparedness and co-ordination in Health Protection.
    b. Provide training and regular updates on governance for staff.
    c. Incorporate a quality improvement programme.
    d. Develop a clear strategic plan including the development of a prioritisation framework to aid transparent decision making and resource allocation.
    e. Ensure clear and transparent Human Resources communication about purpose and function of any new posts created; movement of staff and change of team structures should be risk-assessed.

**5. Create a workplace culture that supports and recognises the value of staff, and their needs**

   a. Proactive planning regarding staff health and wellbeing as part of pandemic response protocols including pacing, task allocation, rest periods, psycho-social supports.
   b. Specifically incorporate surge capacity planning into preparedness plans which are to be reviewed annually.
   c. Undertake formalised, structured situation de-briefs with a focus on staff wellbeing.
   d. Monitor and mitigate the impact of sustained response on staff wellbeing.
   e. Promote gender balance at senior level decision-making fora.

**6. Run effective and efficient meetings**

   a. Provide training for chairing and administration of meetings including agenda setting, appropriate duration, condensed action points and appropriate dissemination of information.
   b. Ensure correct multi-disciplinary representation at meetings as required.
   c. Provide administrative support for all meetings.
   d. Listen to all representatives; respect and value contributions.
   e. Audit meeting effectiveness.

## 14. The Hexagon Tool[5]

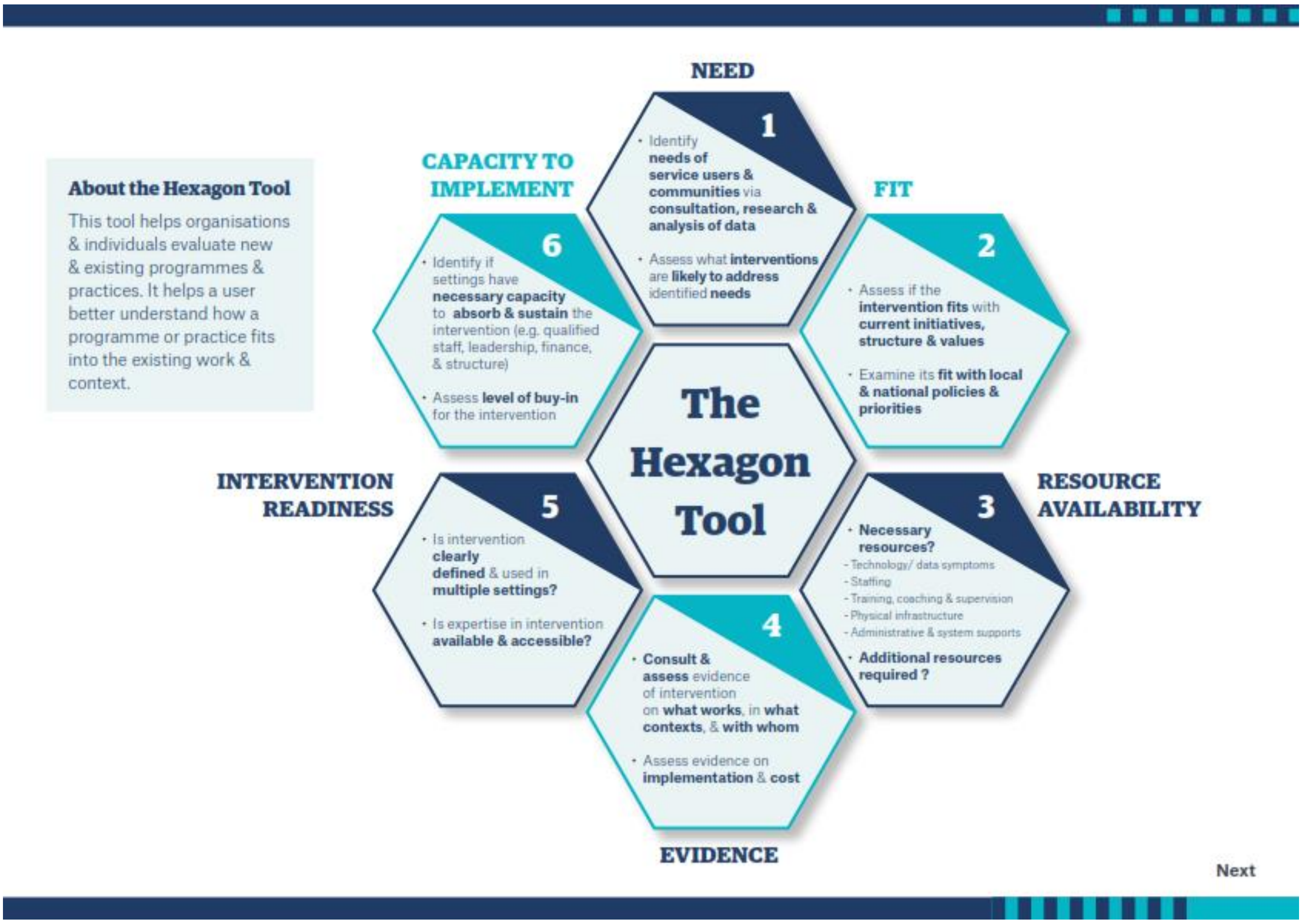


[5] Ward C, Ihlo T, Morgan W. Hexagon tool. Chapel Hill: The University of North Carolina, Frank Porter Graham Child Development Institute; 2019. (Accessed:2 Dec 2022). Available from: https://fpg.unc.edu/publications/hexagon-tool
